# Supplementary material for: A mechanism of origin licensing control through autoinhibition of S. cerevisiae ORC·DNA·Cdc6
Source: Nat Commun. 2022 Feb 25;13:1059. doi: 10.1038/s41467-022-28695-w (PMC8881611; doi:10.1038/s41467-022-28695-w)
Supplement: Supplementary file 1 — Supplementary Information [file 41467_2022_28695_MOESM1_ESM.pdf]

A mechanism of origin licensing control through autoinhibition of *S. cerevisiae* ORC·DNA·Cdc6

Jan Marten Schmidt, Ran Yang, Ashish Kumar, Olivia Hunker, Jan Seebacher, and Franziska Bleichert

Supplementary Information

Supplementary Figures 1-12

Supplementary Table 1

Supplementary References

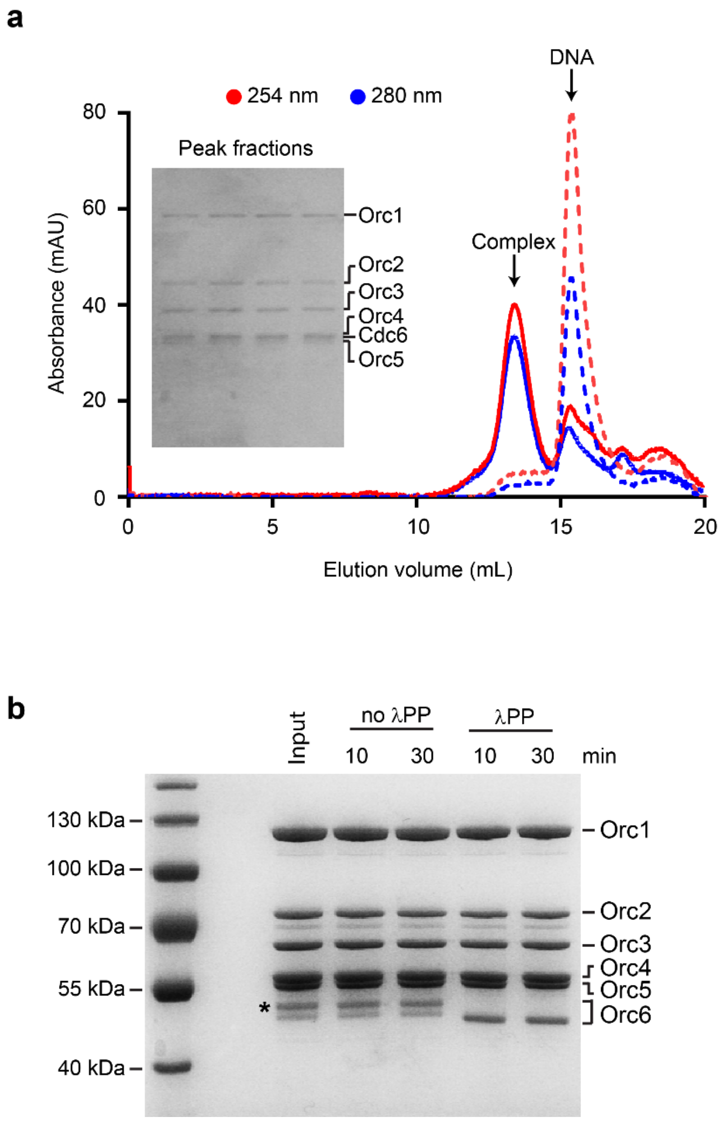

Supplementary Figure 1. Purification of *Sc*ORC·DNA·Cdc6. **a**) Gel filtration chromatography of a reaction containing ARS1 DNA, *Sc*ORC and *Sc*Cdc6 (solid lines) indicates complex formation. A chromatogram of a control reaction containing DNA only (dashed lines) is shown for comparison. Peak fractions contained ORC and Cdc6 when analyzed by SDS-PAGE and Coomassie staining. Note that Coomassie staining was not sensitive enough to resolve Orc6 after gel filtration since it migrates as multiple bands due to differences in phosphorylation status (see **b**). **b**) Orc6 in *Sc*ORC used for ternary complex reconstitution is phosphorylated. Coomassie-stained SDS-PAGE gel of recombinant *Sc*ORC with or without λ phosphatase (λPP) treatment indicates that at least 50% of Orc6 is phosphorylated (marked by an asterisk). Experiments were conducted two or more times. Source data are provided as a Source Data file.

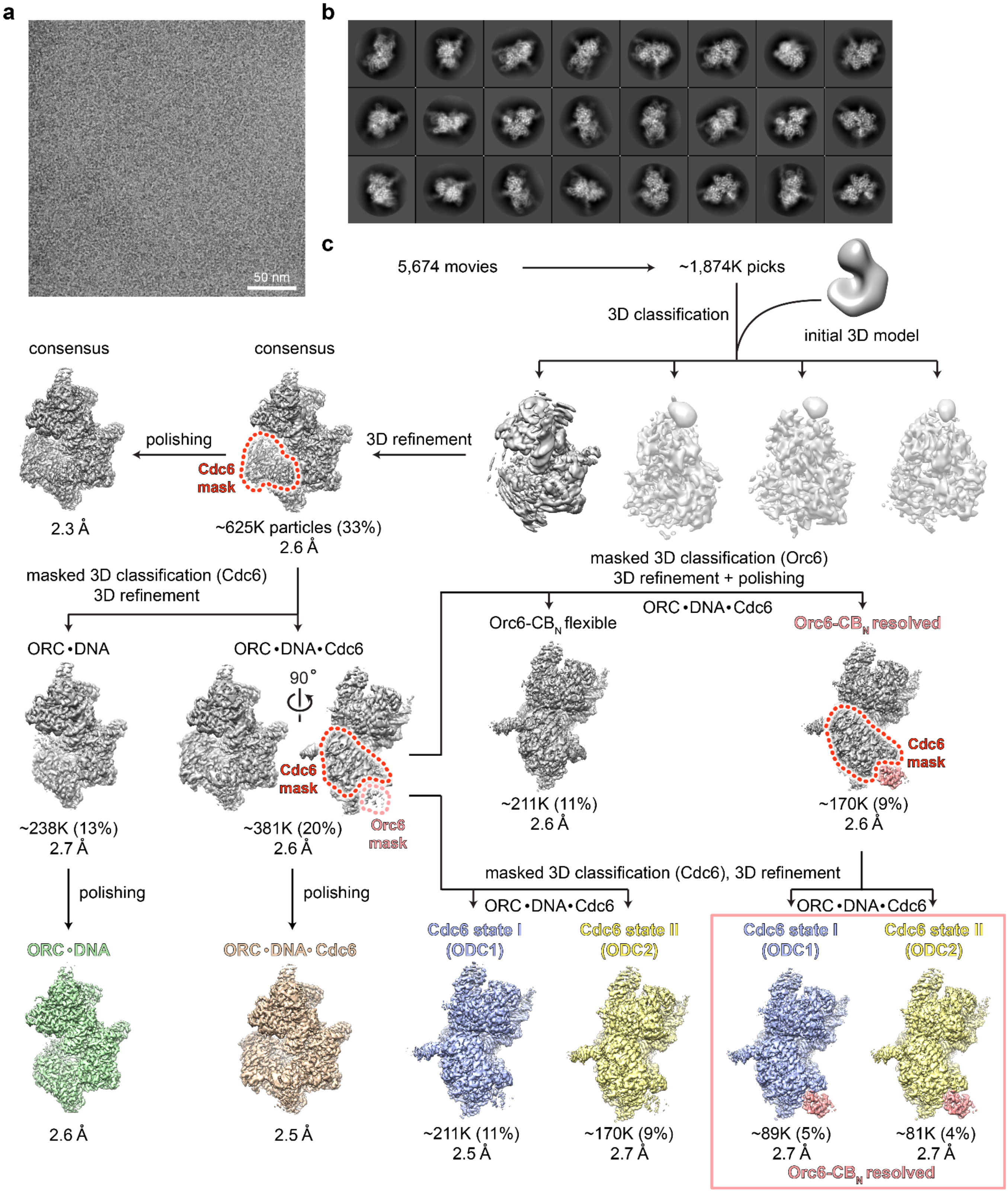

Supplementary Figure 2. Summary of cryo-EM data processing workflow. **a**) Representative image of summed movie frames. **b**) 2D class averages of *Sc*ORC·DNA·Cdc6. **c**) 3D classification and refinement scheme that yielded 2.3-2.7 Å resolution structures for *Sc*ORC·DNA and *Sc*ORC·DNA·Cdc6 assemblies. Masks used for masked classifications are indicated by dotted outlines.

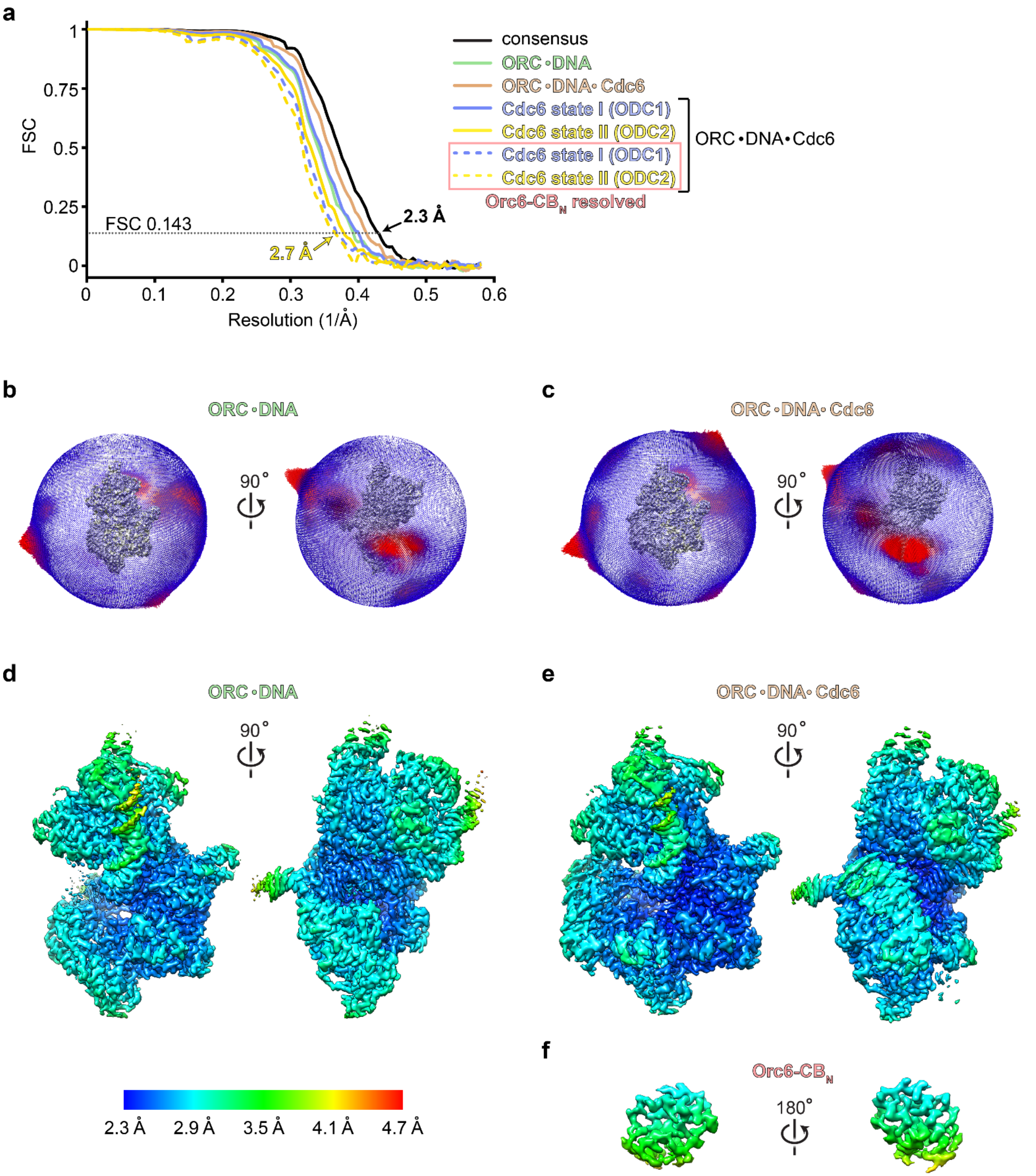

Supplementary Figure 3. Resolutions and angular particle distributions for *Sc*ORC·DNA and *Sc*ORC·DNA·Cdc6 assemblies. **a**) Gold-standard Fourier shell correlation (FSC) curves for reconstructed cryo-EM maps. **b** and **c**) Angular distribution of particles contributing to *Sc*ORC·DNA (in **b**) and *Sc*ORC·DNA·Cdc6 (in **c**) maps. **d-f**) Unsharpened cryo-EM maps of *Sc*ORC·DNA (in **d**), *Sc*ORC·DNA·Cdc6 (consensus, in **e**), and the Orc6-CB$_N$ region (when docked onto the ORC·Cdc6 ring, in **f**) colored by local resolution.

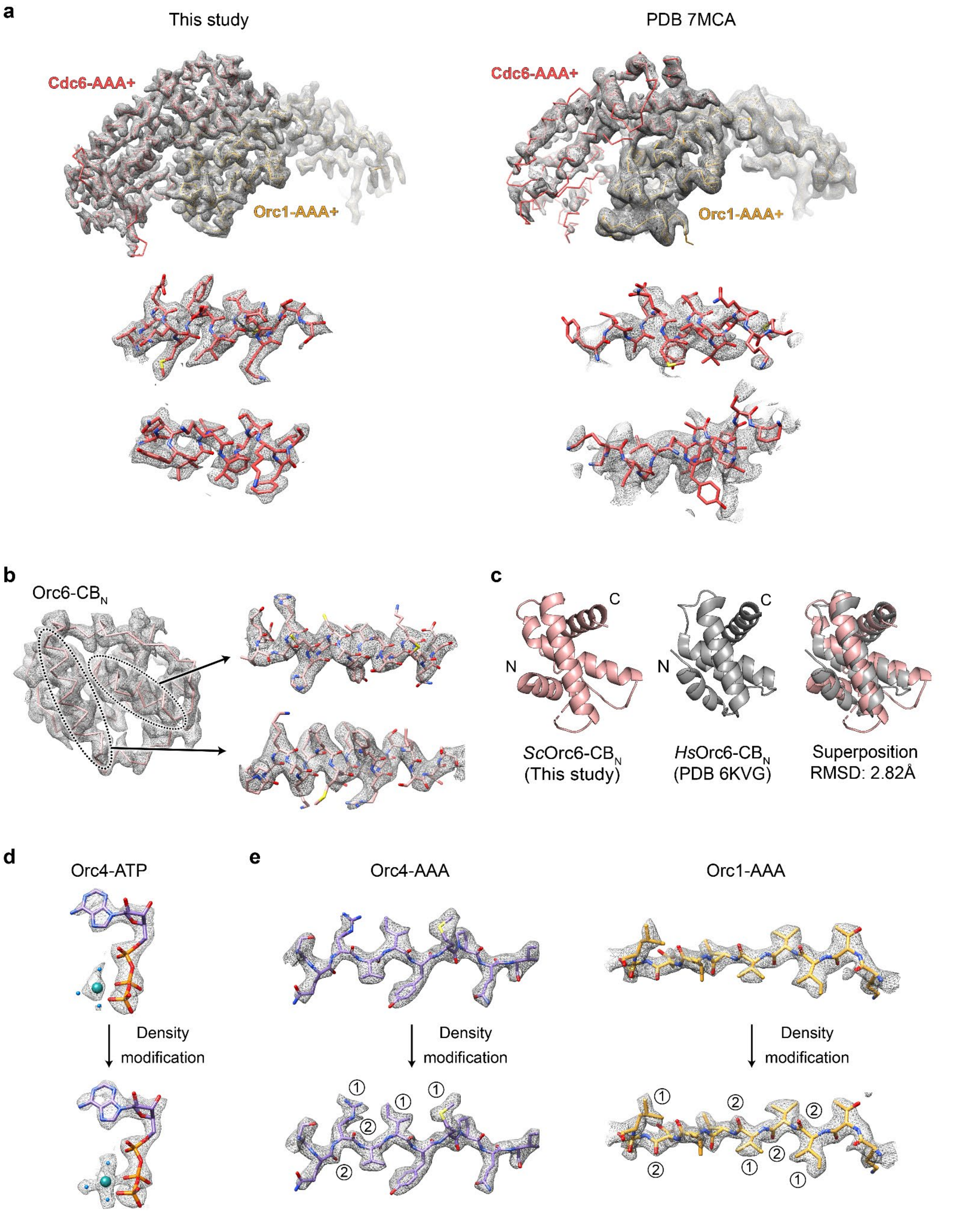

Supplementary Figure 4. Examples of cryo-EM map densities. **a**) Comparison of Orc1 and Cdc6 cryo-EM map regions (unsharpened) of our *Sc*ORC·DNA·Cdc6 structure with a recent lower resolution structure of this

complex (PDB 7mca, EMD-23755[1]) reveals weak Cdc6 density in the earlier study. The unsharpened cryo-EM map for EMD-23755 is not deposited and was therefore calculated from deposited half-maps with RELION. Zoomed views of two different Cdc6 α-helices with corresponding cryo-EM map densities are also shown. **b**) Unsharpened cryo-EM map density and model for Orc6-CB_N. **c**) Structural comparison of *S. cerevisiae* and human (*Hs*) Orc6-CB_N domains (PDB 6kvg[2]). **d** and **e**) Density modification improves cryo-EM map features for water molecules (in **d**), sidechains (in **e**, marked with “1”) and backbone carbonyls (in **e**, marked with “2”).

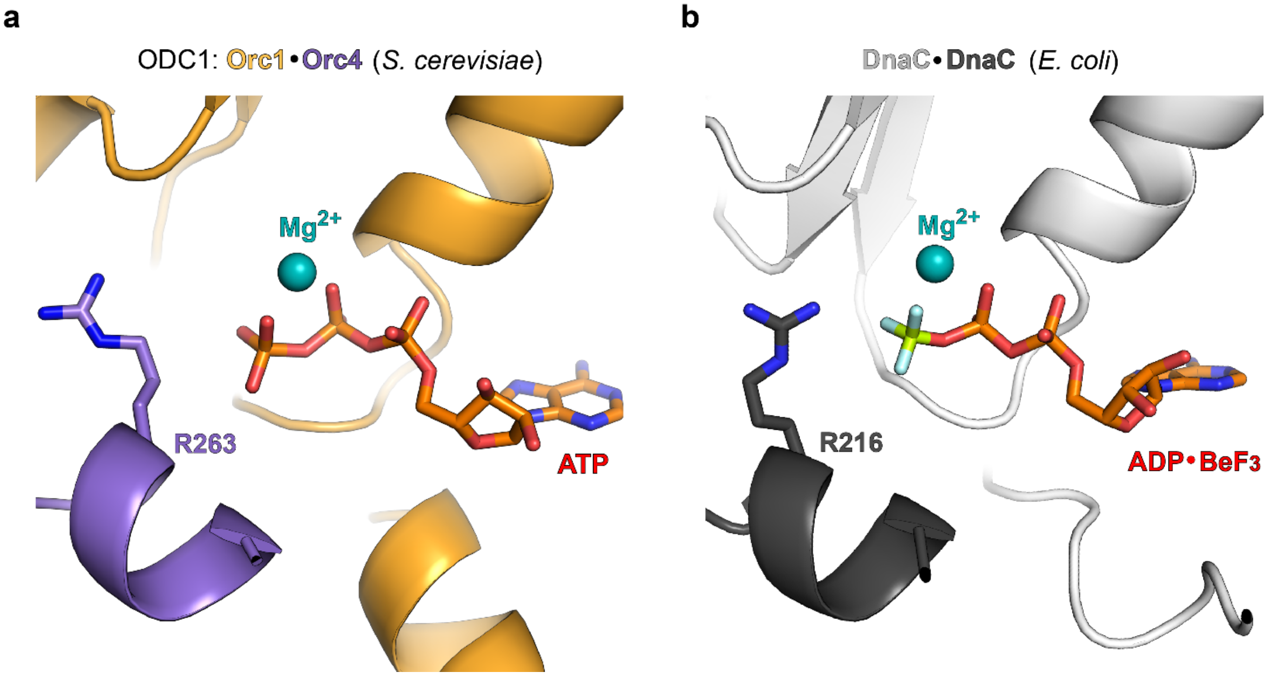

Supplementary Figure 5. Comparison of *Sc*Orc4-R263 tether and *E. coli* DnaC Arg coupler R216. **a**) *S. cerevisiae* Orc1·Orc4 ATPase center of ODC1 with Orc4-R263 shown as stick. **b**) DnaC ATPase site in the *apo* DnaBC complex (PDB 6qel[3]). The Arg coupler R216 is depicted as stick.

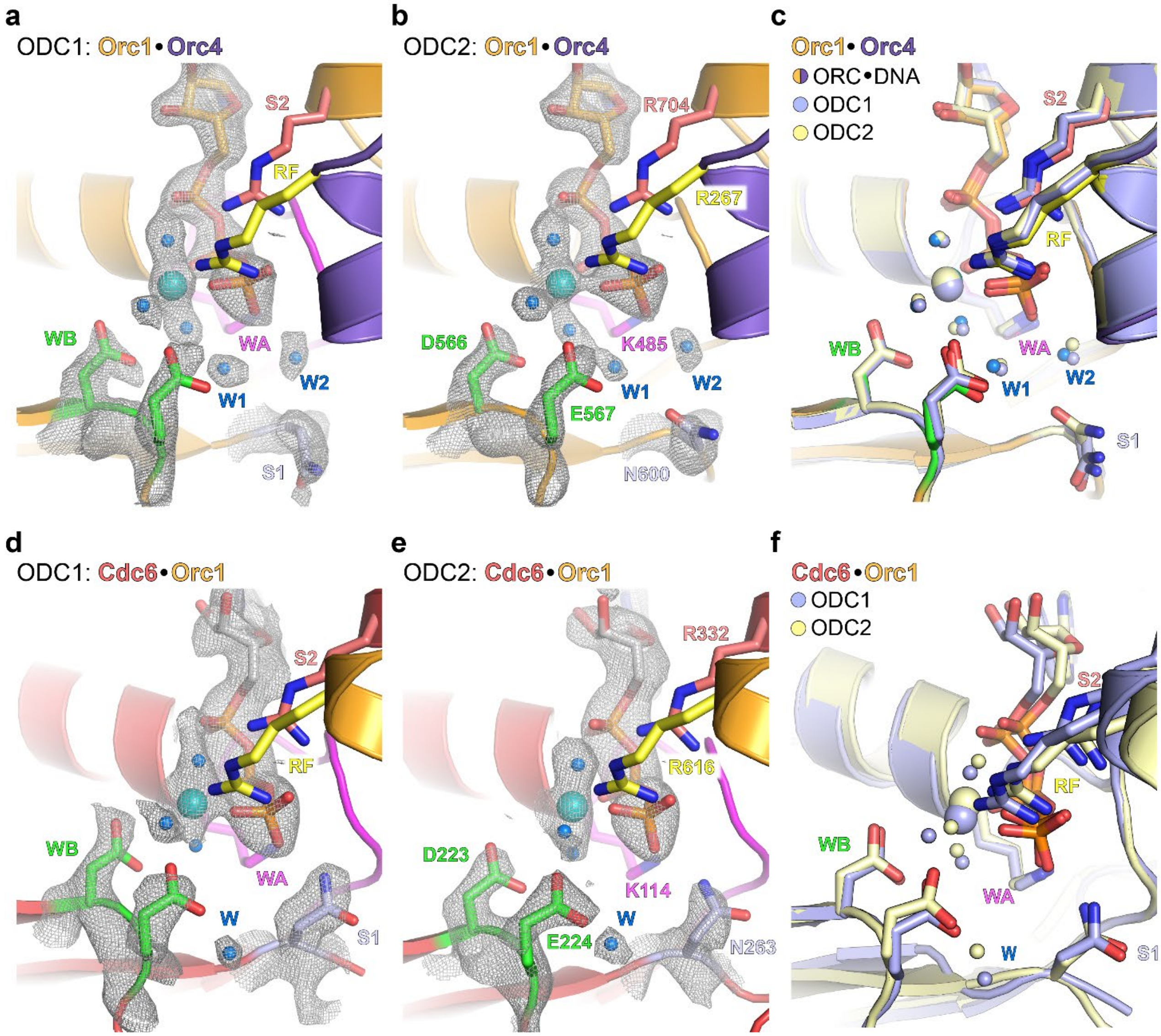

Supplementary Figure 6. Configuration of ATPase sites in *Sc*ORC·DNA·Cdc6. **a** to **c**) Orc1·Orc4 ATPase centers of ODC1 (in **a**), ODC2 (in **b**), and superposed onto the same site in *Sc*ORC·DNA (in **c**). **d-f**) Cdc6·Orc1 ATPase sites of ODC1 (in **d**) and ODC2 (in **e**), and superposed onto each other (in **f**). Weak density for additional waters in non-lytic positions is not shown for clarity in **d-f**. Density-modified cryo-EM maps are shown as grey mesh for ATP, magnesium, water (W) molecules, as well as for Walker B (WB) and sensor 1 (S1) residues. Walker A (WA), Walker B, sensor 1, sensor 2 (S2), and arginine finger (RF) side chains are depicted as sticks. Water and magnesium are shown as blue and teal spheres, respectively, in **a**, **b**, **d**, **e**.

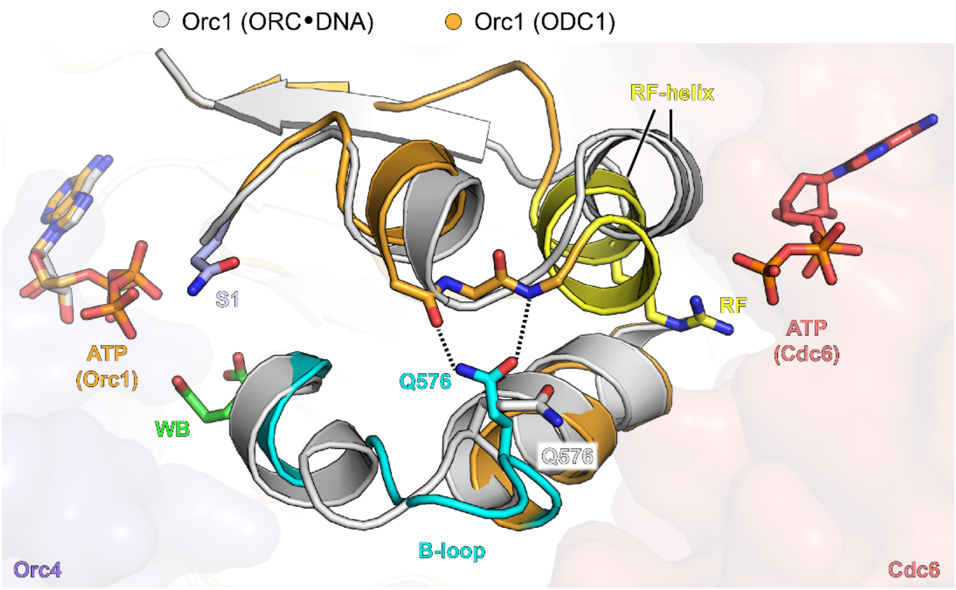

Supplementary Figure 7. A conformational change in the B-loop of Orc1, accompanied by a rotamer switch of Q576, helps reposition the arginine finger helix upon Cdc6 binding. The Orc1 subunit of *Sc*ORC·DNA·Cdc6 (ODC1, in color) and *Sc*ORC·DNA (in grey) are superposed, and regions including and surrounding the Orc1 B-loops and arginine finger helices are shown for both complexes. Dashed lines correspond to hydrogen bonds formed between Q576 (in ODC1) and main chain CO and NH groups of a loop preceding the arginine finger (RF) helix. ATPs bound to Orc1 and Cdc6 are shown in stick representation. Orc4 and Cdc6 are rendered as transparent surface.

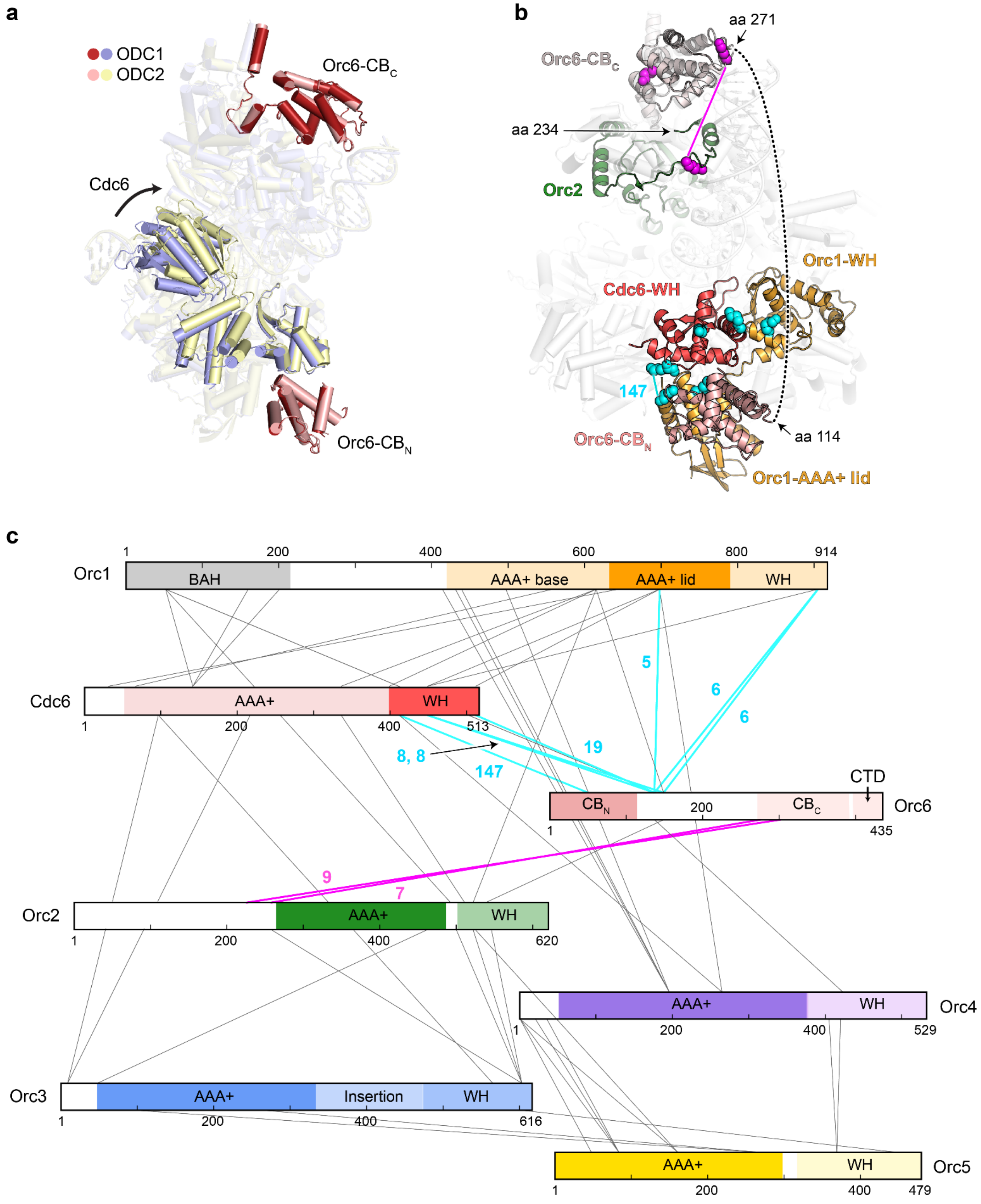

Supplementary Figure 8. Conformational states of *Sc*ORC·DNA·Cdc6 with docked Orc6-CB$_N$ and crosslinking mass spectrometry of the ternary complex. **a**) Docking of Orc6-CB$_N$ onto Orc1 and Cdc6 is observed in both ODC1 and ODC2 states. Models built into cryo-EM maps of the ODC1 and ODC2 ternary complexes with docked Orc6-CB$_N$ were superposed by structural alignment of Orc2 in both assemblies to visualize repositioning of Cdc6.

Cdc6 and Orc6 are shown as solid cartoons, while other subunits are transparent. The conformational change in the ORC·Cdc6 ring is similar to that observed in complexes with flexible Orc6-CB_N (see **Figure 1c**). **b** and **c**) Crosslinking mass spectrometry of *Sc*ORC·DNA·Cdc6. **b**) Lysine residues involved in crosslinks between Orc6-CB_N or the beginning of the disordered linker region (up to residue 150), the Orc1-AAA+ lid, and the WH domains of Orc1 and Cdc6 are shown as cyan spheres. A solid cyan line connects crosslinked lysines in Orc6-CB_N and Cdc6-WH, the most abundant crosslink observed (147 CSM). Crosslinked lysines between Orc6-CB_C and Orc2 are in magenta. Absence of solid lines between crosslinked residues indicates that one of the lysines is located in a disordered region in the structure. The amino acid (aa) residues at the C-terminus of Orc6-CB_N and the N-terminus Orc6-CB_C are marked, as is the first resolved residue in Orc2. **C**) Graphical summary of inter-protein crosslinks in *Sc*ORC·DNA·Cdc6. Crosslinks that report on the location of Orc6-CB_N are highlighted in cyan, while those between the Orc6-CB_C and Orc2 are shown in magenta (see also **Figure 3e**). The number of CSMs for these crosslinks is listed.

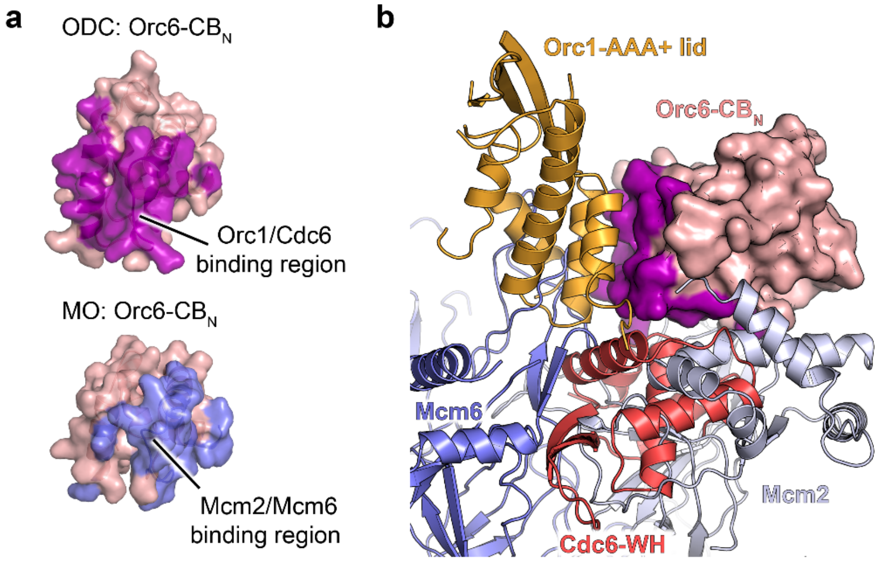

Supplementary Figure 9. Orc1/Cdc6 in *Sc*ORC·DNA·Cdc6 and Mcm2/Mcm6 in the MO complex (PDB 6rqc[4]) occupy similar binding sites on Orc6-CB$_N$. **a**) Orc6-CB$_N$ in *Sc*ORC·DNA·Cdc6 and in the MO complex is shown in surface representation with Orc1/Cdc6 and Mcm2/Mcm6 binding regions colored purple or blue. **b**) Superposition of Orc6-CB$_N$ in MO and ODC reveals severe clashes between Orc1/Cdc6 and Mcm2/Mcm6, indicating that docking of this domain onto ORC·Cdc6 and Mcm2-7 are mutually exclusive. Mcm2, Mcm6, Orc1-AAA+ lid and Cdc6-WH regions are shown as cartoon. Orc6-CB$_N$ is represented as surface with the Orc1/Cdc6 binding site colored purple.

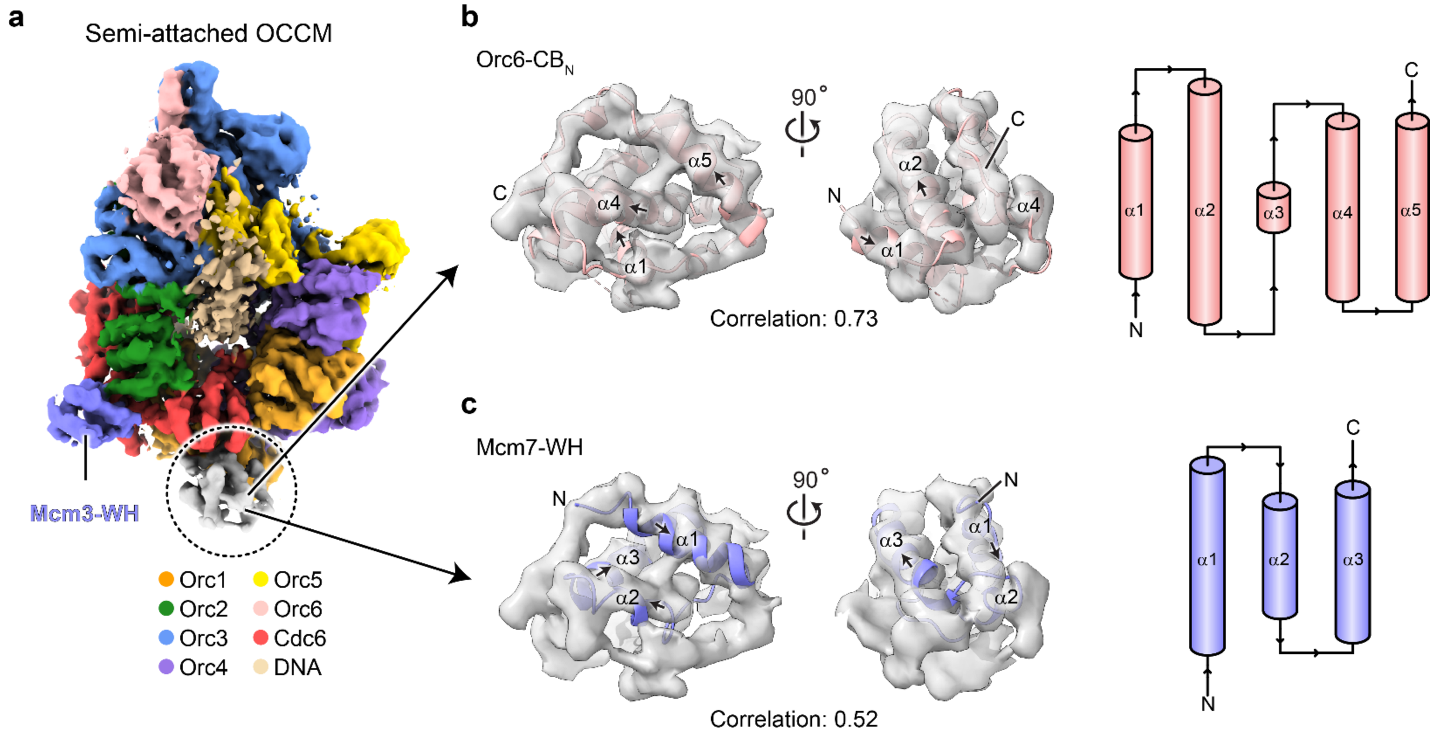

Supplementary Figure 10. The semi-attached OCCM corresponds to a stalled loading intermediate. **a**) Cryo-EM map of the semi-attached OCCM reported previously (EMD-21662[5]) colored by subunit. The regions assigned as the WH domains of Mcm3 (in blue) and Mcm7 (in grey, circled) are indicated. **b** and **c**) Docking of Orc6-CB$_N$ (in **b**, this study) and Mcm7-WH (in **c**, PDB 6wgc[5]) models into the "Mcm7-WH" EM map region of the semi-attached OCCM. The correlation between each model and the EM map at a resolution of 8 Å was calculated with the "Fit in Map" command in ChimeraX. Topology diagrams for Orc6-CB$_N$ and Mcm7-WH models are also shown. The substantially better fit of Orc6-CB$_N$ to the map argues that this region of the semi-attached OCCM complex corresponds to the N-terminal domain of Orc6 and not the WH domain of Mcm7. Orc6-CB$_N$ docking supports Mcm2-7 recruitment to ORC·DNA·Cdc6 through binding of the Mcm3-WH domain, but the blocked binding site for the Mcm7-WH domain would prevent productive engagement of Mcm2-7 for loading.

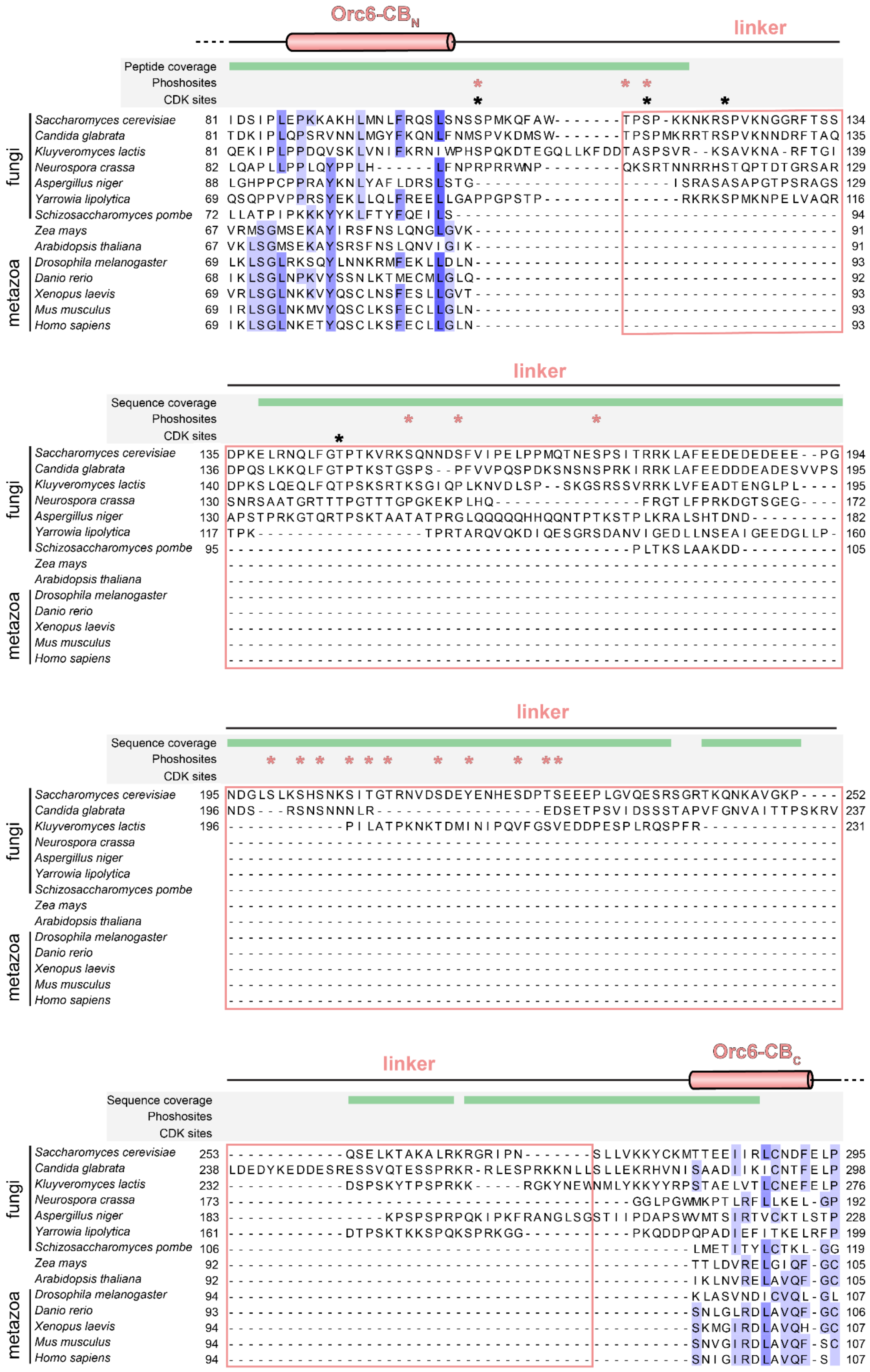
Orc6-CBN
linker
Peptide coverage
Phoshosites
CDK sites
fungi
metazoa
Saccharomyces cerevisiae
Candida glabrata
Kluyveromyces lactis
Neurospora crassa
Aspergillus niger
Yarrowia lipolytica
Schizosaccharomyces pombe
Zea mays
Arabidopsis thaliana
Drosophila melanogaster
Danio rerio
Xenopus laevis
Mus musculus
Homo sapiens
Sequence coverage
Orc6-CBC

Supplementary Figure 11. Multiple protein sequence alignment of fungal, plant, and metazoan Orc6. Known CDK phosphorylation sites in *S. cerevisiae* Orc6 and phosphorylated residues in our ORC preparation with PTM scores > 50 (determined by mass spectrometry) are marked by asterisks above the protein sequence. Mass spectrometry sequence coverage is indicated in green. Only the C-terminal region of Orc6-CB_N and the N-terminal sequence of Orc6-CB_C that are adjacent to the linker insertion are shown, with secondary structure elements included above the sequence.

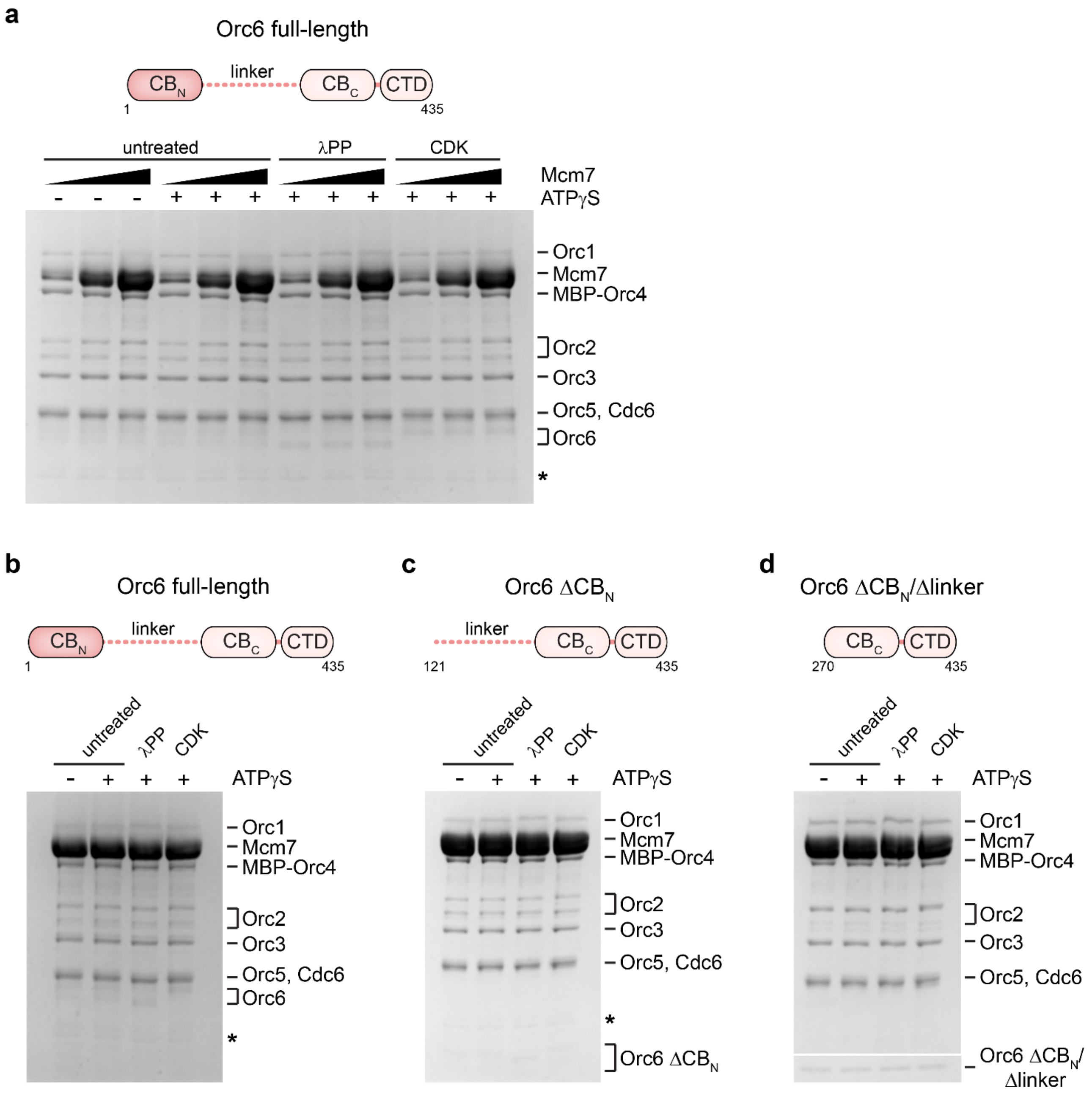

Supplementary Figure 12. Coomassie-stained SDS-PAGE gels of inputs for Mcm7 recruitment assays shown in **Figure 5b-e** (in the same order). Experiments were repeated as indicated in **Figure 5**. Source data are provided as a Source Data file.

Supplementary Table 1. Summary of cryo-EM data collection, refinement, and validation statistics

**Sample**	***Sc*ORC·DNA·Cdc6**
**EM data collection and processing:**	
Microscope	Titan Krios (EFTEM)
Camera	K2 Summit
Voltage (kV)	300
Magnification	x130,000
Frames (no.)	50
Total electron dose (e^{-}/Å^2)	49.3
Electron dose rate (e^{-}/ Å^2/s)	6.6
Calibrated pixel size (Å)	0.86
Defocus range (μm)	-0.8 to -1.6
Micrographs	5,674
Initial picks (no.)	1,873,616

	*Sc*ORC·DNA<br><br>PDB 7TJF<br>EMD-25924	*Sc*ORC·DNA·Cdc6 (ODC1)<br>PDB 7TJH<br>EMD-25925	*Sc*ORC·DNA·Cdc6 (ODC2)<br>PDB 7TJI<br>EMD-25926	*Sc*ORC·DNA·Cdc6·Orc6-CB_N (ODC1)<br>PDB 7TJJ<br>EMD-25927	*Sc*ORC·DNA·Cdc6·Orc6-CB_N (ODC2)<br>PDB 7TJK<br>EMD-25928
Refined particles (no.)	238,056	211,406	170,092	88,859	81,494
Symmetry imposed	C1	C1	C1	C1	C1
Global resolution (Å)					
FSC 0.5 (unmasked/masked)	3.4/3.0	3.4/2.9	3.6/3.0	3.8/3.1	3.9/3.1
FSC 0.143 (unmasked/masked)	3.0/2.6	3.0/2.5	3.1/2.7	3.1/2.7	3.2/2.7
Local resolution range (Å)	2.4 - 4.1	2.4 - 4.1	2.5 - 4.3	2.6 - 4.5	2.6 - 4.7
Map sharpening B factor (Å^2)	-46	-47	-48	-43	-44
**Model refinement and validation:**					
Initial model	5zr1	5zr1	5zr1	5zr1	5zr1
Model composition					
Non-hydrogen atoms	21,125	23,899	23,766	24,732	24,668
Protein residues	2,327	2,689	2,676	2,791	2,785
DNA residues	82	82	82	82	82
Ligands (ATP, Mg)	3, 3	4, 4	4, 4	4, 4	4, 4
Waters	297	148	112	160	130
Root mean square deviation					
Bond lengths (Å)	0.005	0.005	0.005	0.006	0.005
Bond angles (°)	0.868	0.913	0.899	0.942	0.885

	*Sc*ORC·DNA	*Sc*ORC·DNA·Cdc6 (ODC1)	*Sc*ORC·DNA·Cdc6 (ODC2)	*Sc*ORC·DNA·Cdc6·Orc6-CB_N (ODC1)	*Sc*ORC·DNA·Cdc6·Orc6-CB_N (ODC2)
B factors ($Å^2$)					
Protein	76.32	84.13	87.80	81.61	87.51
DNA	91.37	101.27	127.29	103.79	116.46
Ligands	58.64	63.46	58.52	56.94	57.87
Water	60.38	62.43	60.63	58.93	59.89
Ramachandran plot					
% favored	97.85	97.04	96.98	97.11	97.13
% allowed	2.15	2.96	3.02	2.89	2.87
% outliers	0.0	0.0	0.0	0.0	0.0
Rotamer outliers (%)	1.87	2.06	2.52	0.47	0.39
MolProbity					
Clashscore	4.00	3.36	4.51	3.95	3.81
MolProbity score	1.43	1.54	1.71	1.34	1.33
Model-map comparison					
EM Ringer score	3.79	3.79	3.53	4.0	3.6
CC_{mask}	0.89	0.88	0.88	0.87	0.87
$FSC_{model/map}$ 0.5	2.6	2.6	2.7	2.8	2.8

Supplementary References

1. Feng, X. et al. The structure of ORC-Cdc6 on an origin DNA reveals the mechanism of ORC activation by the replication initiator Cdc6. *Nat Commun* **12**, 3883 (2021).

2. Xu, N. et al. Structural basis of DNA replication origin recognition by human Orc6 protein binding with DNA. *Nucleic Acids Res* **48**, 11146-11161 (2020).

3. Arias-Palomo, E., Puri, N., O'Shea Murray, V.L., Yan, Q. & Berger, J.M. Physical Basis for the Loading of a Bacterial Replicative Helicase onto DNA. *Mol Cell* **74**, 173-184 (2019).

4. Miller, T.C.R., Locke, J., Greiwe, J.F., Diffley, J.F.X. & Costa, A. Mechanism of head-to-head MCM double-hexamer formation revealed by cryo-EM. *Nature* **575**, 704-710 (2019).

5. Yuan, Z. et al. Structural mechanism of helicase loading onto replication origin DNA by ORC-Cdc6. *Proc Natl Acad Sci U S A* **117**, 11747-17756 (2020).
