# Supplementary material for: A mechanism of origin licensing control through autoinhibition of S. cerevisiae ORC·DNA·Cdc6
Source: Nat Commun. 2022 Feb 25;13:1059. doi: 10.1038/s41467-022-28695-w (PMC8881611; doi:10.1038/s41467-022-28695-w)
Supplement: Supplementary file 3 — Reporting Summary [file 41467_2022_28695_MOESM3_ESM.pdf]

nature portfolio

Corresponding author(s): Franziska Bleichert

Last updated by author(s): Jan 29, 2022

Reporting Summary

Nature Portfolio wishes to improve the reproducibility of the work that we publish. This form provides structure for consistency and transparency in reporting. For further information on Nature Portfolio policies, see our Editorial Policies and the Editorial Policy Checklist.

Statistics

For all statistical analyses, confirm that the following items are present in the figure legend, table legend, main text, or Methods section.

n/a	Confirmed	
☐	☒	The exact sample size (*n*) for each experimental group/condition, given as a discrete number and unit of measurement
☐	☒	A statement on whether measurements were taken from distinct samples or whether the same sample was measured repeatedly
☒	☐	The statistical test(s) used AND whether they are one- or two-sided<br>*Only common tests should be described solely by name; describe more complex techniques in the Methods section.*
☒	☐	A description of all covariates tested
☒	☐	A description of any assumptions or corrections, such as tests of normality and adjustment for multiple comparisons
☐	☒	A full description of the statistical parameters including central tendency (e.g. means) or other basic estimates (e.g. regression coefficient) AND variation (e.g. standard deviation) or associated estimates of uncertainty (e.g. confidence intervals)
☒	☐	For null hypothesis testing, the test statistic (e.g. *F*, *t*, *r*) with confidence intervals, effect sizes, degrees of freedom and *P* value noted<br>*Give P values as exact values whenever suitable.*
☒	☐	For Bayesian analysis, information on the choice of priors and Markov chain Monte Carlo settings
☒	☐	For hierarchical and complex designs, identification of the appropriate level for tests and full reporting of outcomes
☒	☐	Estimates of effect sizes (e.g. Cohen's *d*, Pearson's *r*), indicating how they were calculated

Our web collection on statistics for biologists contains articles on many of the points above.

Software and code

Policy information about availability of computer code

Data collection	EPU 2.3.0.20
Data analysis	MotionCor2 20170130, GCTF v1.06, GAUTOMATCH 0.53, RELION 2.1 and 3.0, PHENIX 1.19.2_4158, COOT 0.9.6, MAFFT v7.427, PyMOL 2.1.0, UCSF Chimera 1.14, UCSF ChimeraX 1.2.5 , Jalview 2.10.5

For manuscripts utilizing custom algorithms or software that are central to the research but not yet described in published literature, software must be made available to editors and reviewers. We strongly encourage code deposition in a community repository (e.g. GitHub). See the Nature Portfolio guidelines for submitting code & software for further information.

Data

Policy information about availability of data

All manuscripts must include a data availability statement. This statement should provide the following information, where applicable:

- Accession codes, unique identifiers, or web links for publicly available datasets
- A description of any restrictions on data availability
- For clinical datasets or third party data, please ensure that the statement adheres to our policy

The PDB coordinates and cryo-EM maps have been deposited into the Protein Data Bank and Electron Microscopy Data Band under the following accession numbers: PDB 7TJF and EMD-25924 for ScORC·DNA, PDB 7TJH and EMD-25925 for ScORC·DNA·Cdc6 state I (ODC1), PDB 7TJI and EMD-25926 for ScORC·DNA·Cdc6 state II (ODC2), PDB 7TJJ and EMD-25927 for ScORC·DNA·Cdc6·Orc6-CBN state I (ODC1·Orc6-CBN), and PDB 7TJK and EMD-25928 for ScORC·DNA·Cdc6·Orc6-CBN state II (ODC2·Orc6-CBN). The proteomics data have been submitted to the ProteomeXchange Consortium via the PRIDE partner repository with the dataset identifier PXD031033.

March 2021

Field-specific reporting

Please select the one below that is the best fit for your research. If you are not sure, read the appropriate sections before making your selection.

☒ Life sciences ☐ Behavioural & social sciences ☐ Ecological, evolutionary & environmental sciences

For a reference copy of the document with all sections, see nature.com/documents/nr-reporting-summary-flat.pdf

Life sciences study design

All studies must disclose on these points even when the disclosure is negative.

Sample size	Sample size of the cryo-EM dataset is included in Table 1 and in the processing flowchart in the Supplemental Figures. The sample size was not pre-determined but was dependent on the available microscope time and the desired resolutions of the final cryo-EM maps.
Data exclusions	Cryo-EM movies with high drift and low resolution estimation were excluded since they would negatively impact the resolution of the final cryo-EM reconstructions. Damaged particles or wrongly-picked particles were excluded from final maps by 2D and 3D classification. This exclusion procedure is common practice in cryo-EM data processing to maximize the final map resolution.
Replication	Crosslinking mass spectrometry was performed at three different concentrations of crosslinker, each repeated twice in independent experiments. Biochemical experiments were successfully repeated two or more times.
Randomization	Cryo-EM particles were randomly split into separate datasets during 3D refinement to allow resolution estimation using the gold-standard fourier shell correlation. Additional randomization of data is not relevant to this study.
Blinding	Blinding is not relevant to this study.

Reporting for specific materials, systems and methods

We require information from authors about some types of materials, experimental systems and methods used in many studies. Here, indicate whether each material, system or method listed is relevant to your study. If you are not sure if a list item applies to your research, read the appropriate section before selecting a response.

Materials & experimental systems

n/a	Involved in the study	
☒	☐	Antibodies
☐	☒	Eukaryotic cell lines
☒	☐	Palaeontology and archaeology
☒	☐	Animals and other organisms
☒	☐	Human research participants
☒	☐	Clinical data
☒	☐	Dual use research of concern

Methods

n/a	Involved in the study	
☒	☐	ChIP-seq
☒	☐	Flow cytometry
☒	☐	MRI-based neuroimaging

Eukaryotic cell lines

Policy information about cell lines

Cell line source(s)	Thermo Fischer Scientific, High Five cells (BTI-TN-5B1-4) and Sf9 cells (Spodoptera frugiperda)
Authentication	Cell lines were not authenticated.
Mycoplasma contamination	Cell lines were not checked for mycoplasma contamination.
Commonly misidentified lines (See ICLAC register)	No commonly misidentified cell lines were used in the study.
